# Whole-transcriptome analysis of UUO mouse model of renal fibrosis reveals new molecular players in kidney diseases

**DOI:** 10.1038/srep26235

**Published:** 2016-05-18

**Authors:** Eleni Arvaniti, Panagiotis Moulos, Athina Vakrakou, Christos Chatziantoniou, Christos Chadjichristos, Panagiotis Kavvadas, Aristidis Charonis, Panagiotis K. Politis

**Affiliations:** 1 Center for Clinical, Experimental Surgery and Translational Research, Biomedical Research Foundation of the Academy of Athens, Athens, Greece; 2 HybridStat Predictive Analytics, Athens, Greece; 3 INSERM UMR-S1155, Tenon Hospital, Paris, France; 4 Center for Basic Research, Biomedical Research Foundation of the Academy of Athens, Athens, Greece

## Abstract

Transcriptome analysis by RNA-seq technology allows novel insights into gene expression and regulatory networks in health and disease. To better understand the molecular basis of renal fibrosis, we performed RNA-seq analysis in the Unilateral Ureteric Obstruction (UUO) mouse model. We analysed sham operated, 2- and 8-day post-ligation renal tissues. Thousands of genes with statistical significant changes in their expression were identified and classified into cellular processes and molecular pathways. Many novel protein-coding genes were identified, including critical transcription factors with important regulatory roles in other tissues and diseases. Emphasis was placed on long non-coding RNAs (lncRNAs), a class of molecular regulators of multiple and diverse cellular functions. Selected lncRNA genes were further studied and their transcriptional activity was confirmed. For three of them, their transcripts were also examined in other mouse models of nephropathies and their up- or down-regulation was found similar to the UUO model. *In vitro* experiments confirmed that one selected lncRNA is independent of TGFβ or IL1b stimulation but can influence the expression of fibrosis-related proteins and the cellular phenotype. These data provide new information about the involvement of protein-coding and lncRNA genes in nephropathies, which can become novel diagnostic and therapeutic targets in the near future.

Chronic kidney disease (CKD) is a frequent condition, causing severe long-term effects with devastating personal and societal consequences^1^,^2^,^3^. There is a need for novel approaches to prevent the decline in renal function during progression of CKD. Considering that the structural basis for this decline is the development of fibrosis, we believe that understanding the molecular basis of renal fibrosis, could offer valuable insights for the improvement of monitoring techniques and therapeutic interventions.

To address this question, we combined a systems biology approach in animal models for renal fibrosis, focusing on (but not limited to) the unilateral ureteric obstruction (UUO) model^4^,^5^. We identified the full transcriptome of renal tissue, using the RNA-seq methodology, during early and late time intervals of kidney fibrosis. This methodology allows the identification of new protein-coding transcripts and novel non-coding RNA transcripts^6^. This is an exciting new direction, since about 75% of the mammalian genome (including human) is transcribed but not translated into proteins, and certain types of non-coding RNAs, especially long non coding RNAs (lncRNAs), play critical regulatory roles in many biological processes^7^,^8^. However, no data are currently available on the full transcriptome analysis of renal tissue from the UUO model in mice. By performing whole transcriptome sequencing and thorough bioinformatics analysis, we gathered novel information regarding up-regulated and down-regulated genes, pathways and biological processes, and we made lists of differentially expressed genes not suspected so far to be involved in the process of renal fibrosis and differentially expressed lncRNAs. Furthermore, we showed that selected lncRNAs are also differentially expressed in other renal pathology models (two chronic ones exhibiting fibrosis and one acute with no fibrosis), and overexpression of these lncRNAs is sufficient to cause functional changes in a kidney cell line.

Overall, we describe, for the first time, the involvement of a class of lncRNA and protein-coding genes in renal dysfunction, raising the exciting prospect of utilizing this knowledge for better understanding renal pathologies and development of new diagnostic and therapeutic tools.

## Results

**Figure 1:**
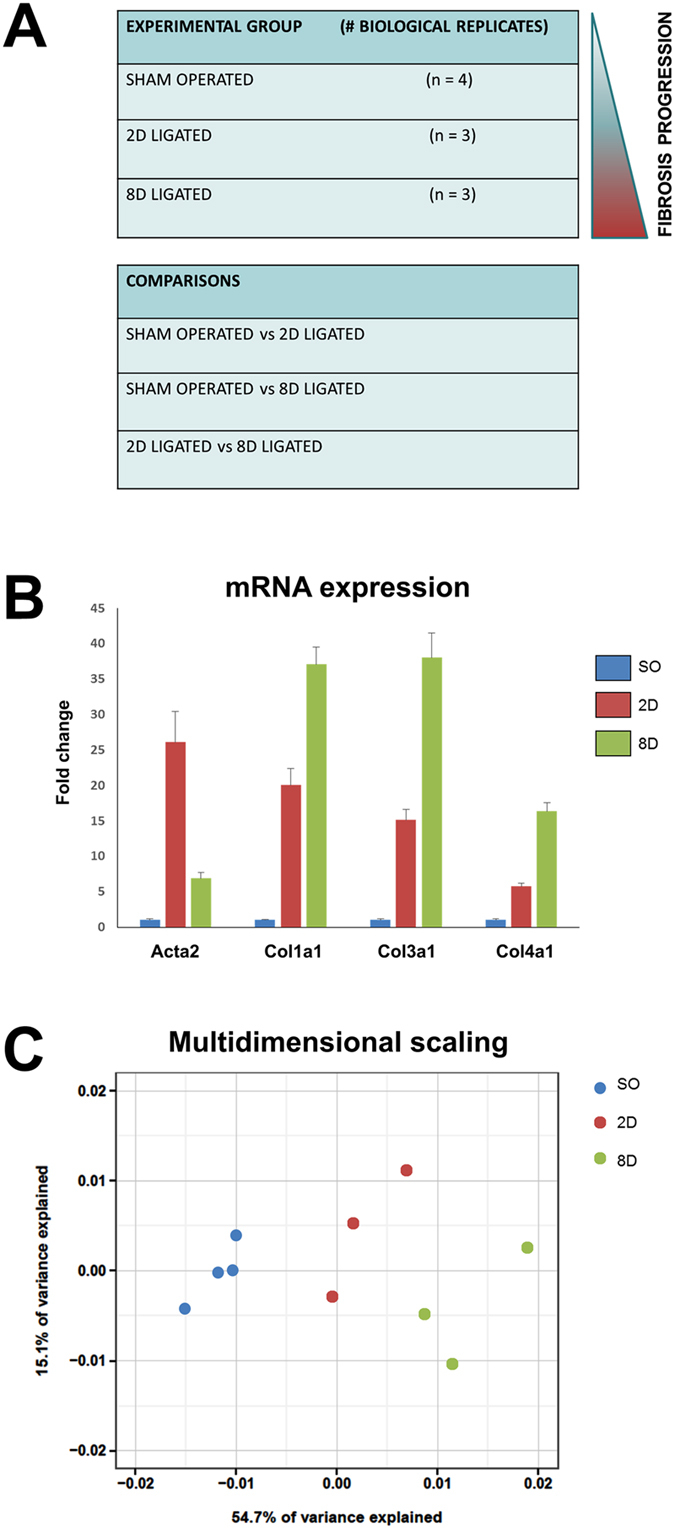
(**A**) Experimental material and biological replicates used in the comparisons of the cohort. (**B**) Verification of the mRNA expression of genes known to be affected in renal fibrosis. The mRNA levels of each gene were normalized to GAPDH and expressed as fold of induction/change compared to sham operated animals. Acta2: Alpha smooth muscle actin, Col1a1: Collagen alpha-1 type I, Col3a1: Collagen alpha-1 type III, Col4a1: Collagen alpha-1 type IV. (**C**) Multidimentional scaling analysis. Confirmation of the high correlation and reproducibility among the samples of each group SO: Sham operated, 2D: 2D ligated and 8D: 8D ligated.

To identify new molecular players in renal fibrosis, high throughput RNA-seq was used in the mouse UUO model. Kidneys of 6 UUO mice (time intervals 2 and 8 days post-ligation) and 4 Sham operated mice (Fig. 1A) were harvested and total RNA was used as input to generate Illumina TrueSeq libraries. Prior to RNA-seq analysis, RNA samples and tissue samples were analyzed to confirm molecular changes indicative of the fibrotic signature (Fig. 1B; Supplemental Fig. 1 and data not shown^9^). Libraries were sequenced, low-quality reads and rRNA sequences were filtered, total clean reads were mapped to genome and mapped reads were assembled into putative transcripts (Supplemental Table 1). The number of detected genes per sample as defined by RPKM values (reads per kilobase of exon per million reads) are reported in Supplemental Table 2, while the mean number of identified genes per group, defined by the same means, were 18790, 19572 and 20061 for the Sham Operated, 2D ligated and 8D ligated groups respectively. These data have been deposited in NCBI’s Gene Expression Omnibus^10^,^11^ and are accessible through GEO Series accession number GSE79443. (https://www.ncbi.nlm.nih.gov/geo/query/acc.cgi?acc=GSE79443).

### Identification of differentially expressed genes during progression of renal fibrosis

**Figure 2:**
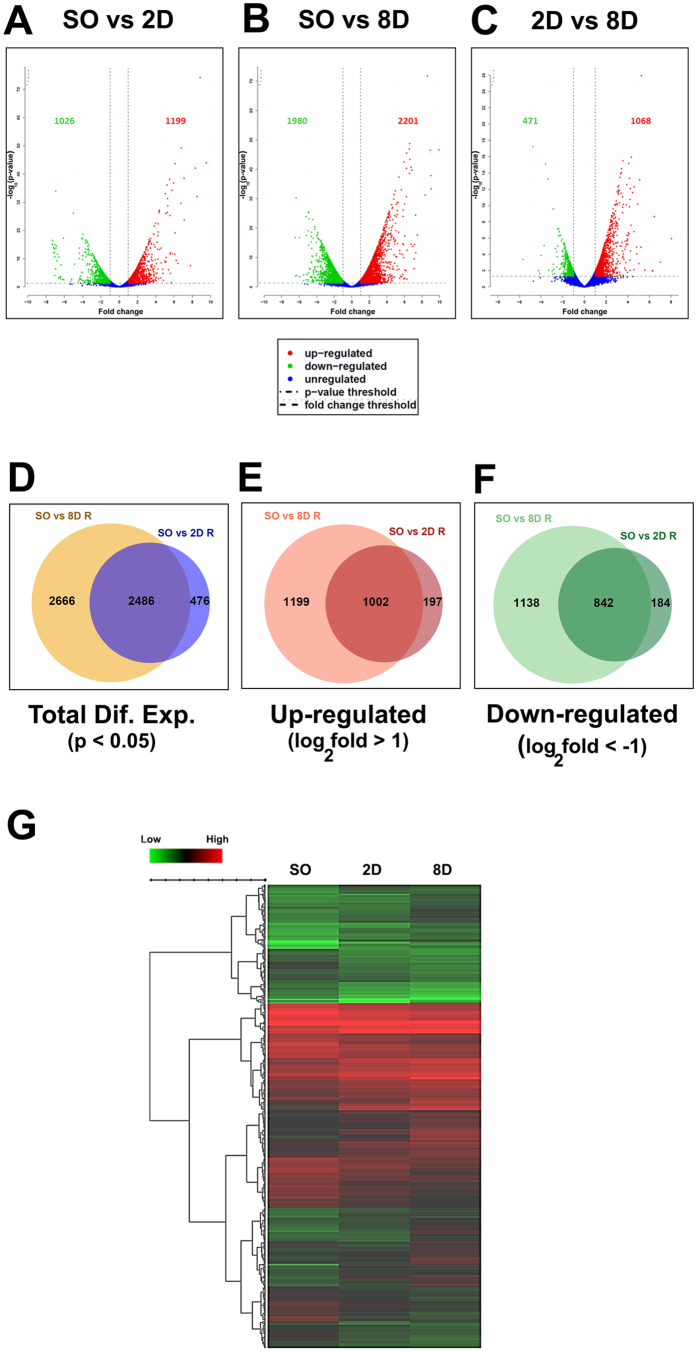
Differentially expressed genes in the UUO model of renal fibrosis. (**A**) Volcano plot for the comparison between Sham Operated (SO) and 2D ligated (2D) samples. Only the statistically significant genes are represented in the graph. In all cases, red color is indicative of up-regulated and green color of down-regulated genes. Blue color indicates genes that even though are differentially expressed in statistical significant manner they do not pass the cutoff values of 1 and −1 in log_2_ scale (>2 folds up-regulation and <0.5 folds down-regulation, respectively) that were utilized to identify up-regulated and down-regulated genes, respectively. The cutoffs are schematically represented as vertical lines in the volcano plots of (**A–C**). (**B**) Volcano plots for the comparison between Sham Operated (SO) and 8D ligated (8D) samples. Only the statistically significant genes are represented in the graphs. (**C**) Volcano plot for the comparison between 2D ligated (2D) and 8D ligated (8D) samples. Only the statistically significant genes are represented in the graphs. (**D**) Total statistically significant differentially expressed genes (Dif. Exp.) in the comparisons between Sham operated vs 2D ligated (SO vs 2D) and Sham operated vs 8D ligated (SO vs 8D). (**E**) Up-regulated statistically significant differentially expressed genes in the comparisons between Sham operated vs 2D ligated (SO vs 2D) and Sham operated vs 8D ligated (SO vs 8D) as defined by the cut-off value of >1 log_2_ fold change. (**F**) Down-regulated statistically significant differentially expressed genes in the comparisons between Sham operated vs 2D ligated (SO vs 2D) and Sham operated vs 8D ligated (SO vs 8D) as defined by the cut-off value of <−1 log_2_ fold change. (**G**) Hierarchical clustering (Euclidean distance, Ward linkage) and heatmap showing the expression changes between Sham operated, 2D ligated, 8D ligated, expressed as the log2 of normalized and summed exonic read counts in Sham operated, 2D ligated and 8D ligated samples.

Multidimensional scaling analysis confirmed high correlation and reproducibility among individual samples of each group (Fig. 1C). The bioinformatics analysis was focused on three independent comparisons: 2 days ligated vs Sham operated (SO vs 2D), 8 days ligated vs Sham operated (SO vs 8D) and 8 days ligated vs 2 days ligated (2D vs 8D). Statistically significant (p < 0.05), differentially expressed genes are reported in detail (Supplemental Tables 3–5). We identified 2962 genes differentially expressed in SO vs 2D, 5152 genes in SO vs 8D and 1854 genes in 2D vs 8D. An absolute fold change cutoff value of 1 in log_2_ scale was utilized to identify up-regulated and down-regulated genes, respectively, in each independent comparison (cutoffs represented as vertical lines in volcano plots of Fig. 2). Venn diagrams were used to schematically represent similarities between SO vs 2D and SO vs 8D (Fig. 2D–F). Many genes were found only in one of the two comparisons, indicating differential temporal expression (Fig. 2D–F). Hierarchical clustering (Euclidean distance, Ward linkage) and heatmaps (Fig. 2G) show the expression changes between SO, 2D and 8D. The depicted genes exhibit statistically significant differential expression (Benjamini-Hochberg FDR < 0.05) in at least one of the comparisons. Fig. 2A–C,G are available as interactive links online (http://epigenomics.fleming.gr/metaseqr_runs/HS/000005sub/ and http://epigenomics.fleming.gr/inchlib_heatmaps/HS/BRFAA/Charonis/val_005_r/).

### Identification of cellular processes, molecular pathways and specific genes involved in renal fibrosis

Gene Ontology (GO) and pathway analyses were carried out to determine molecular processes and biological pathways associated with differentially expressed genes in all comparisons. We associated differentially expressed mRNAs with two structured networks provided by the Genecodis website: biological process (BP) and molecular function (MF) (Supplementary Tables 6–17). The GO analysis further validated our experimental set up, by demonstrating that many biological processes and molecular functions, already associated with fibrosis, are also enriched in our datasets, including cell adhesion, inflammatory response, apoptosis, cell proliferation, cell differentiation, proteolysis, etc (Supplementary Tables 6–17). Interestingly, we were able to identify a large number of individual genes in these categories that have not been previously correlated with renal fibrosis or kidney pathologies, even though the specific GO category/term is strongly associated with this disease. Only in the up-regulated genes for GO term: Cell Adhesion, in SO vs 2D comparison (Supplementary Table 6), we were able to identify 13 genes with very important biochemical/biological functions in other tissues or organs. The list of genes includes *Fblim1*, *Epha2*, *Wisp1*, *Parvg*, *Madcam1*, *Mybpc2*, *Cdh3*, *Gpr56*, *Troap*, *Pstpip1*, *Pcdh8*, *Nlgn2* and *Mfap4,* genes that based on Pubmed and Kidney and Urinary Pathway Knowledge Base^12^ searches have not been previously associated with renal fibrosis. Their importance is significant as: For example, Fblim1 is involved in the assembly and stabilization of actin-filaments and plays a role in modulating cell adhesion, cell morphology and cell motility^13^,^14^. Epha2 belongs to the ephrin receptor subfamily of the protein-tyrosine kinase family and regulates cellular morphology, adhesion and migration^15^,^16^. Wisp1 is a member of the WNT1 inducible signaling pathway and localized in the extracellular matrix, where it acts to regulate adhesion, proliferation and apoptosis^17^,^18^,^19^. All these genes and many more in the other GO categories or comparisons (Supplementary Tables 6–17) constitute an extremely rich source for potential target genes in understanding and combating renal fibrosis.

In the same comparison by screening the GO categories containing the term transcriptional regulation, we identified 42 genes encoding for transcription factors or regulators not previously associated with renal fibrosis (as defined by searches in Pubmed and kupkb) that were up-regulated in SO vs 2D comparison; most of these factors were also up-regulated in SO vs 8D comparison, indicative of consistent actions and/or functional roles (Table 1). Many of these factors play critical roles in other organs’ development and homeostasis and in human diseases, suggesting similar roles in renal pathophysiology. Collectively, these data offer a long list of novel candidate genes with potential implications in renal fibrosis. For example, Sox9, which is induced by 55.1 folds in SO vs 2D and 50.4 in SO vs 8D, is a HMG-box class transcription factor and a master regulator of the pancreatic program, liver progenitors, duodenal development, chondrogenesis, sex determination, Sertoli cell differentiation during testis development, craniofacial development and is mutated in campomelic dysplasia, a disorder characterized by skeletal malformations, XY sex reversal, and neonatal lethality^20^,^21^,^22^. Runx1, which is induced by 5.1 folds in SO vs 2D and 18.2 in SO vs 8D, is a heterodimeric transcription factor that binds to the core element of many enhancers and promoters, with important regulatory functions in hematopoietic stem/progenitor cells and causal roles in human leukemias as well as solid tumors^23^,^24^. Uhrf1, induced 11.4 folds in SO vs 2D and 11.5 in SO vs 8D, and Ezh2 (member of the Polycomp repressive complex), induced 2.3 folds in SO vs 2D and 2.01 in SO vs 8D, are both central epigenetic regulators that control chromatin structure and re-organization upon various stimuli. Most importantly these factors regulate gene expression program and cellular behavior, including cell proliferation, migration, differentiation, identity and malignant transformation^25^,^26^,^27^. These observations support a potential role of these factors in early chromatin re-organization events upon induction of fibrotic processes in the kidney. Furthermore, several other critical transcription factors have been found to be strongly up-regulated, including immediate early transcription factors, Fosl1, Fosl2, Fos, Jun, JunD, Egr2, Creb5, as well as other factors such as FoxJ1, Ikzf4, Atf5, Sox4 and Sox11. Collectively, these data offer a long list of novel candidate genes for potential implication in renal fibrosis. Interestingly, for many of these proteins, pharmacological agonists and antagonists have been recently developed, raising exciting possibilities for new therapeutic approaches.

**Figure 3:**
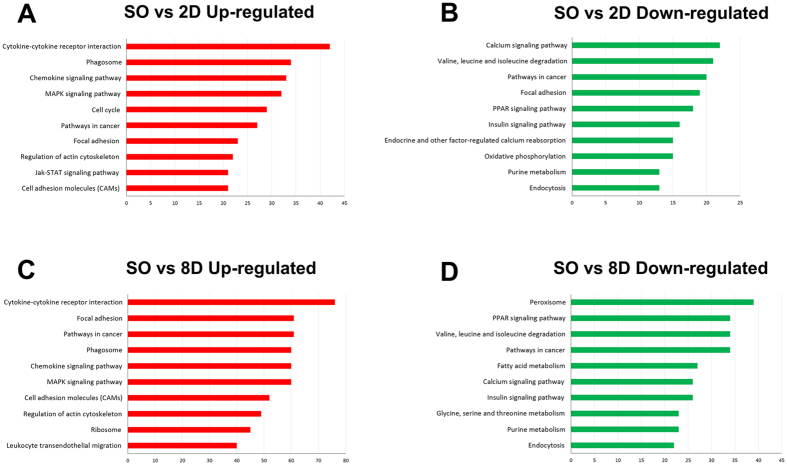
Pathway analysis of differentially expressed genes using GeneCodis. Top 10 of the predominant up-regulated pathways when comparing Sham operated vs 2D ligated samples (SO vs 2D up-regulated) Top 10 of the predominant down-regulated pathways when comparing Sham operated vs 2D ligated samples (SO vs 2D up-regulated) Top 10 of the predominant up-regulated pathways when comparing Sham operated vs 8D ligated samples (SO vs 8D up-regulated) Top 10 of the predominant down-regulated pathways when comparing Sham operated vs 8D ligated samples (SO vs 8D up-regulated).

Next, by pathway analysis we documented a number of biological pathways characterized as up- or down-regulated in all three comparisons. The predominant 25 up- or down- regulated pathways when comparing Sham operated vs ligated samples are summarized in Supplemental Tables 18–23, including corresponding genes, and top 10 of them in Fig. 3A–D.

### Long non-coding RNAs (lncRNAs) are differentially expressed in the UUO animal model

**Figure 4:**
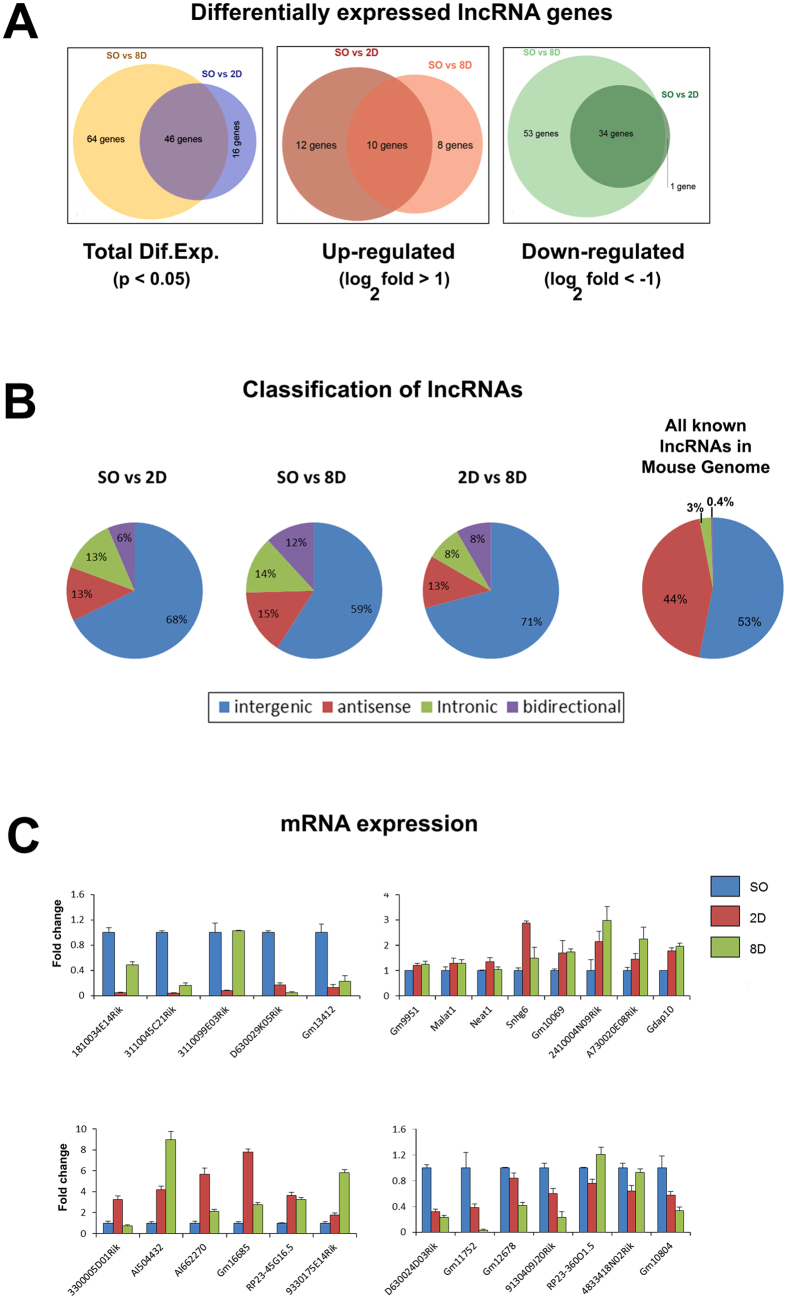
Differentially expressed lncRNAs in the UUO model of renal fibrosis. (**A**) Statistically significant differentially expressed lncRNAs in the comparisons made between Sham operated vs 2D ligated (SO vs 2D) and Sham operated vs 8D ligated (SO vs 8D). (i) Total statistically significant differentially expressed (Dif. Exp.) lncRNAs, (ii) Up-regulated statistically significant differentially expressed (Dif. Exp.) lncRNAs and (iii) Down-regulated statistically significant differentially expressed (Dif. Exp.) lncRNAs. (**B**) Classification of the differentially expressed lncRNAs in the cohort based on their position to the genome and their relationship with protein coding genes. Distribution of all known lncRNAs in the mouse genome. (**C**) Verification of the expression profiles of 26 lncRNAs with real time RT-qPCR analysis. The mRNA levels of each lncRNA were normalized to GAPDH and expressed as fold of induction/change compared to sham operated animals. SO: Sham operated, 2D: 2D ligated and 8D: 8D ligated.

Many lncRNA transcripts were either up-regulated or down-regulated during progression of renal fibrosis. Although lncRNAs consist one of the most abundant classes of RNAs, they remain poorly characterized and their roles in renal physiology or diseases are largely elusive. We identified a long list of differentially expressed lncRNA genes in a statistical significant manner (p < 0.05) (Fig. 4 and Supplementary Tables 24–26). In particular, we identified 62 lncRNAs that were differentially expressed in SO vs 2D, 110 lncRNAs in SO vs 8D and 24 in 2D vs 8D (Fig. 4A).

Next, lncRNA genes were classified according to their relationship with protein-coding genes in their genomic loci (intronic, bidirectional, intergenic, sense/antisense), since this correlation might have functional importance. Details of the distribution of the lncRNAs are schematically presented in Fig. 4B. The majority of the identified lncRNAs, in all comparisons, were intergenic.

Proximity of a lncRNA gene to a protein-coding gene may be a factor contributing to regulatory mechanisms. Therefore, we first performed extensive bioinformatics searches 100 kb upstream and 100 kb downstream of each of these lncRNA genes for the identification of protein-coding genes. These searches were based on the publicly available data of the UCSC genome browser. The examination of the lncRNA’s genomic loci revealed a large number of protein-coding genes that could be potentially affected in cis by the differential expression of the corresponding lncRNAs (listed in Supplemental Tables 27 and 28). In addition, we evaluated these protein-coding genes for similar or opposite patterns of expression with that of corresponding lncRNAs during progression of UUO model, based on our RNA-Seq data. Interestingly, we identified a large number of protein-coding genes with similar or opposite patterns of expression in the genomic vicinity of our differentially expressed lncRNAs (also enlisted in Supplemental Tables 27 and 28).

**Figure 5:**
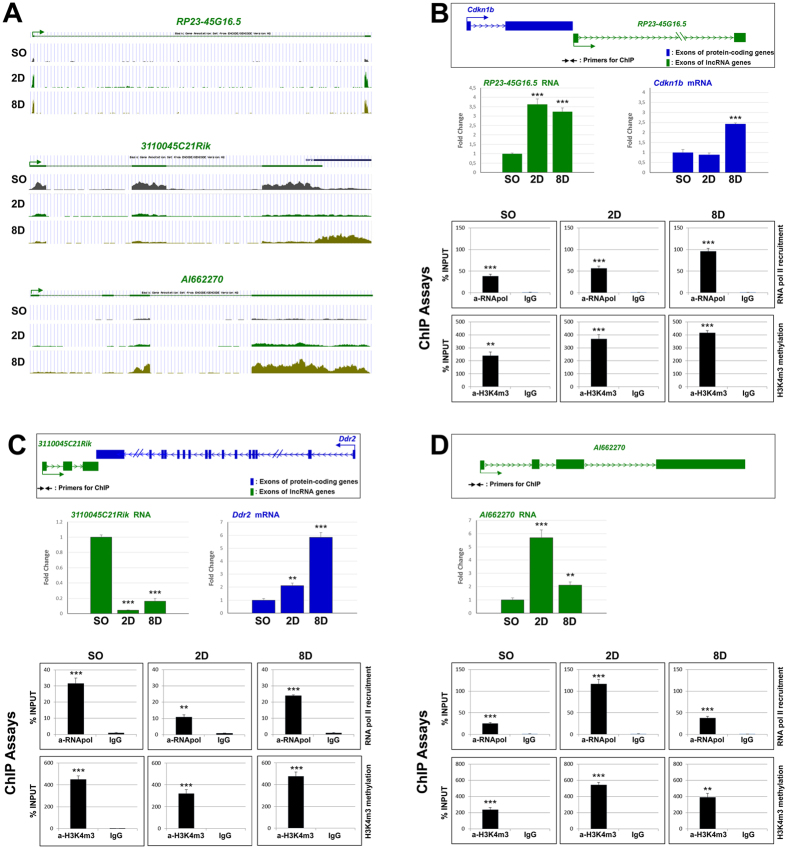
Analysis of selected lncRNAs and their neighboring protein coding genes at both RNA and chromatin organization levels. (**A**) Visualization of RNA-seq peaks for the selected lncRNAs using the UCSC Genome Browser in representative Sham operated (SO), 2D ligated (2D) and 8D ligated (8D) samples (**B**). The example of RP23-45G16.5: (i) Schematic representation of the relative position of the RP23-45G16.5 lncRNA (green color) and its neighboring protein-coding gene Cdkn1b (blue color) in the genome, (ii) mRNA expression analysis of RP23-45G16.5 and its neighboring gene Cdkn1b, as indicated. The mRNA levels of each gene were normalized to GAPDH and expressed as fold of induction/change to sham operated animals, and (iii) ChIP analysis for the binding of RNA polymerase II (RNA pol) and H3K4m3 in the promoter region of RP23-45G16.5. For these experiments, chromatin samples were prepared from kidneys of sham operated (SO), 2 days ligated (2D) and 8 days ligated (8D) mice. *p < 0.05; **p < 0.01, ***p < 0.005, n.s: p = 0.05 (t test), n = 3. (**C**) The example of 3110045C21Rik: (i) Schematic representation of the relative position of the 3110045C21Rik lncRNA (green color) and its neighboring protein-coding gene Ddr2 (blue color) in the genome, (ii) mRNA expression analysis of 3110045C21Rik and its neighboring gene Ddr2, as indicated. The mRNA levels of each gene were normalized to GAPDH and expressed as fold of induction/change to sham operated animals, and (iii) ChIP analysis for the binding of RNA polymerase II (RNA pol) and H3K4m3 in the promoter region of 3110045C21Rik. For these experiments chromatin samples were prepared from kidneys of sham operated (SO), 2 days ligated (2D) and 8 days ligated (8D) mice. *p < 0.05; **p < 0.01, ***p < 0.005, n.s: p = 0.05 (t test), n = 3. (**D**) The example of AI662270: (i) Schematic representation of the position of AI662270 in the genome, (ii) mRNA expression analysis of AI662270 lncRNA. The mRNA levels of AI662270 were normalized to GAPDH and expressed as fold of induction/change to sham operated animals, and (iii) ChIP analysis for the binding of RNA polymerase II (RNA pol) and H3K4m3 in the promoter region of AI662270. For these experiments chromatin samples were prepared from kidneys of sham operated (SO), 2 days ligated (2D) and 8 days ligated (8D) mice. *p < 0.05; **p < 0.01, ***p < 0.005, n.s: p = 0.05 (t test), n = 3.

Following this analysis, and based on proximity of lncRNAs to protein-coding genes with important functions in kidney and/or other tissues as well as on the fold change in their expression levels, we focused on 26 lncRNAs for further analysis. We have chosen the distance of less than 3 kb as a criterion of proximity, mainly due to the general observation that several regulatory elements lay on distances less than 3 kb from their target genes. Regarding the importance, we first sorted out the protein-coding genes in close proximity to our differentially expressed lncRNAs and then by literature search we focused our analysis on genes that were associated with renal pathologies. Expression patterns were analyzed by real-time RT-qPCR (Fig. 4C) and expression levels and fold changes were found in good agreement with the RNA-seq results (Fig. 4C). Although in some cases RNA variation in fold changes was different between real time RT-qPCR and RNA-seq, the expression patterns were coincident between these two techniques (Supplemental Table 29). These data suggest that all 26 lncRNAs are transcribed in renal tissue and their changes in expression levels are either positively or negatively associated with renal pathology in the UUO model. Chromatin immunoprecipitation experiments (ChIP) with antibodies against RNA pol-II and tri-methylation of the fourth lysine (K) residue from the N-terminus of histone H3 (H3K4me3), were performed to specifically evaluate the promoters of lncRNA genes for RNA pol-II recruitment and H3K4me3, which is tightly associated with transcriptional start sites of actively transcribed genes. This analysis was performed for 10 out of the 26 lncRNAs. In all cases, recruitment of RNA pol-II and H3K4me3 were verified for the promoters of these lncRNAs in renal tissue (3 selected cases are depicted in Fig. 5 and the other 7 in Supplemental Figure 2). In most cases the changes in the expression profile were nicely associated with changes in RNA pol-II recruitment and levels of H3K4me3. In detail, in 6/10 cases (more specifically the cases of 3110045C21Rik, AI662270, RP23-45G16.5, 3110099E03Rik, 330005D01Rik and D630024D03Rik) the changes in the expression profile (up- or down- regulation compared to Sham operated) at both 2D- and 8D-interval were followed by similar changes in RNA pol-II recruitment and levels of H3K4me3. In particular, when a lncRNA was characterized as up-regulated in the 6 cases mentioned we also recorded increased recruitment of RNA-pol II and levels of H3K4me3.

Next, the functional role of 3 of these lncRNAs was investigated. We focused on *RP23-45G16.5* transcript, due to its close genomic association with *Cdkn1b* gene encoding for p27-Kip1 regulator of the cell cycle^28^,^29^ (Fig. 5A,B), *3110045C21Rik*, due to its close genomic association with *Ddr2* gene with critical roles in ECM and fibrosis^30^,^31^ (Fig. 5A,C), and *AI662270* due to an early up-regulation in 2D interval (Fig. 5A,D). Interestingly, the changes in the expression profile of *RP23-45G16.5* lncRNA show a positive correlation with the associated protein-coding gene (*Cdkn1b*) (Fig. 5B), while the profile of *3110045C21Rik* shows a negative correlation (Fig. 5C).

### Changes in the expression levels of *RP23-45G16.5*, *3110045C21Rik* and *AI662270* lncRNAs are correlated with renal pathology in other animal models for kidney diseases

**Figure 6:**
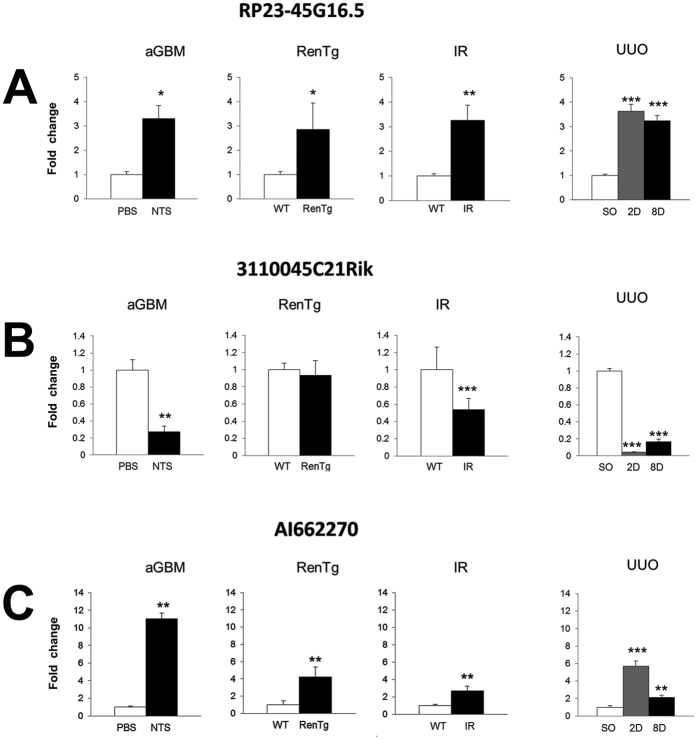
Changes in the expression levels of selected lncRNAs in other animal models for kidney diseases. (**A**) Real time RT-qPCR analysis of the expression profile of RP23-45G16.5 in other models of renal pathology, as indicated. (**B**) Real time RT-qPCR analysis of the expression profile of 3110045C21Rik in other models of renal pathology, as indicated. (**C**) Real time RT-qPCR analysis of the expression profile of AI662270 in other models of renal pathology, as indicated. In all cases: aGBM: anti-GBM model; RenTg: Renin overexpression model; IR: Ischemia/reperfusion model; UUO: Unilateral Ureter Obstruction model. The mRNA levels of each gene were normalized to GAPDH and expressed as fold of induction/change to sham operated animals. *p < 0.05; **p < 0.01, ***p < 0.005, n.s: p = 0.05 (t test), n = 3.

The expression levels of the three selected lncRNAs were further evaluated in three additional animal models (anti-GBM, renin overexpression and ischemia/reperfusion). In the anti-GBM model, the expression levels were evaluated in SV129 wild type female mice, 8 days after the administration of PBS and/or nephrotoxin (NTS). Changes in the expression profiles of the lncRNAs followed similar pattern with that of the UUO (Fig. 6A–C).

In the renin overexpression model, Sv129 10 month-old male mice were used in both wild type (WT) and RenTg background. In two of the lncRNAs cases (AI662270 and RP23-45G16.5) the expression profiles were very similar with those in the UUO model while for the case of 3110045C21Rik no change was observed (Fig. 6).

In the ishemia/reperfusion model, Sv129 male mice subjected to nephrectomy and ischemia for 35 min and reperfusion after 48 h (IR) and wild type (WT) sham operated mice were used. All three lncRNAs expression profiles were very similar to the UUO model (Fig. 6). Collectively, these observations suggest possible involvement of these three lncRNAs in renal pathologies and therefore suggest a more general role for them in kidney diseases.

### *RP23-45G16.5*, *3110045C21Rik* and *AI662270* lncRNAs affect the molecular properties and proliferation of M1 kidney cell line

**Figure 7:**
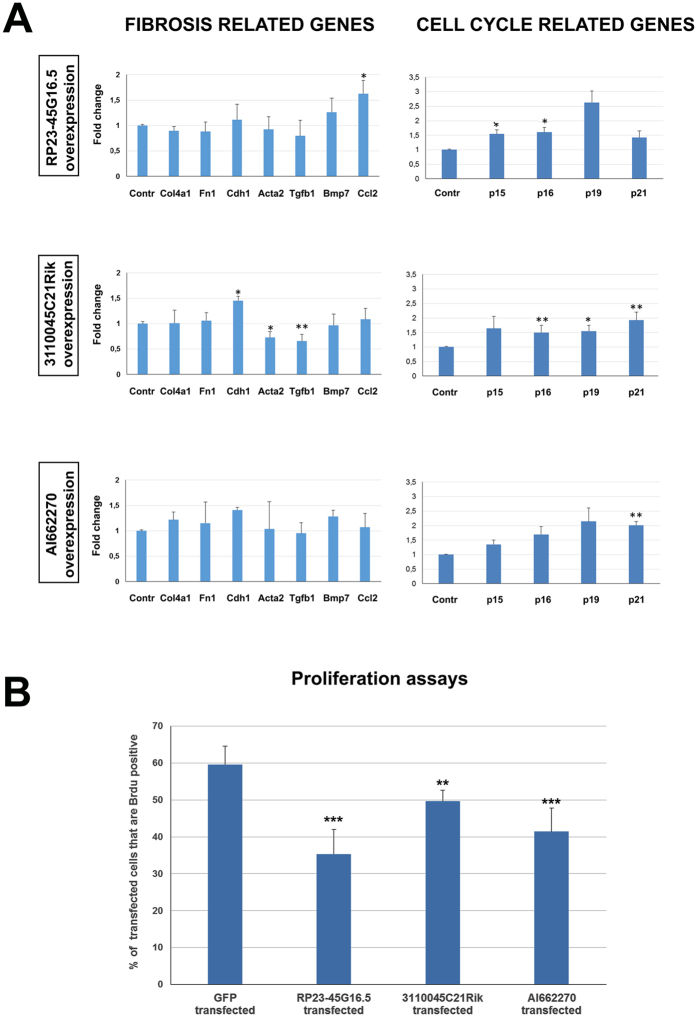
Analysis of the molecular properties and proliferation ability of M1 kidney cell line after overexpression of RP23-45G16.5, 3110045C21Rik and AI662270 lncRNAs. (**A**) mRNA expression profile of genes related to fibrosis or cell cycle in RP23-45G16.5 overexpression, 311045C21Rik overexpression and AI662270 overexpression, as indicated. The mRNA levels of all genes of interest were normalized to GAPDH and expressed as fold of induction/change to control experiment where un-transfected cells were used. *p < 0.05; **p < 0.01, ***p < 0.005, n.s: p = 0.05 (t test), n = 3. (**B**) Proliferation assays performed after co-transfection of cells with a GFP construct and plasmid constructs of the lncRNAs RP23-45G16.5, 311045C21Rik and AI662270. Results were expressed as percentage of the ratio of BrdU measurements (proliferating cells) to GFP measurement (efficiently transfected cells). **p < 0.01, ***p < 0.005, n.s: p = 0.05 (t test), n = 5.

Based on their involvement in many diverse nephropathy models, we explored the putative functional significance of the altered expression of these 3 lncRNAs. Towards this goal, specific phenotypic changes induced by their overexpression were examined in cultured tubular epithelial cells, using the M1 murine cell line, focusing on a panel of fibrosis-related and cell cycle-related genes. The overexpression of the lncRNAs was evaluated by RT-qPCR (Supplemental Figure 3). We show that each lncRNA affects the expression of selected fibrosis-related genes in a different fashion: the overexpression of *RP23-45G16.5* leads to up-regulation of *Ccl2* (chemokine (C-C motif) ligand 2, a pro-inflammatory factor), the overexpression of *3110045C21Rik* leads to up-regulation of *Cdh1* (E-cadherin) and down-regulation of *Acta2* (alpha-SMA) and *Tgfb1* (Transforming Growth Factor, Beta 1), while overexpression of *AI662270* has no statistically significant effect on these genes (Fig. 7A). Regarding cell cycle-related genes, several genes whose products are inhibitors of the cell cycle are significantly up-regulated (Fig. 7A). This finding is further supported by the results from proliferation assays (Fig. 7B), which indicate that at least in our cell system, all three lncRNAs lead to reduction of their proliferative capacity, without affecting cell death (data not shown). Overall, these findings provide evidence that the differential expression of these lncRNAs may exert an important influence on cellular phenotype.

**Figure 8:**
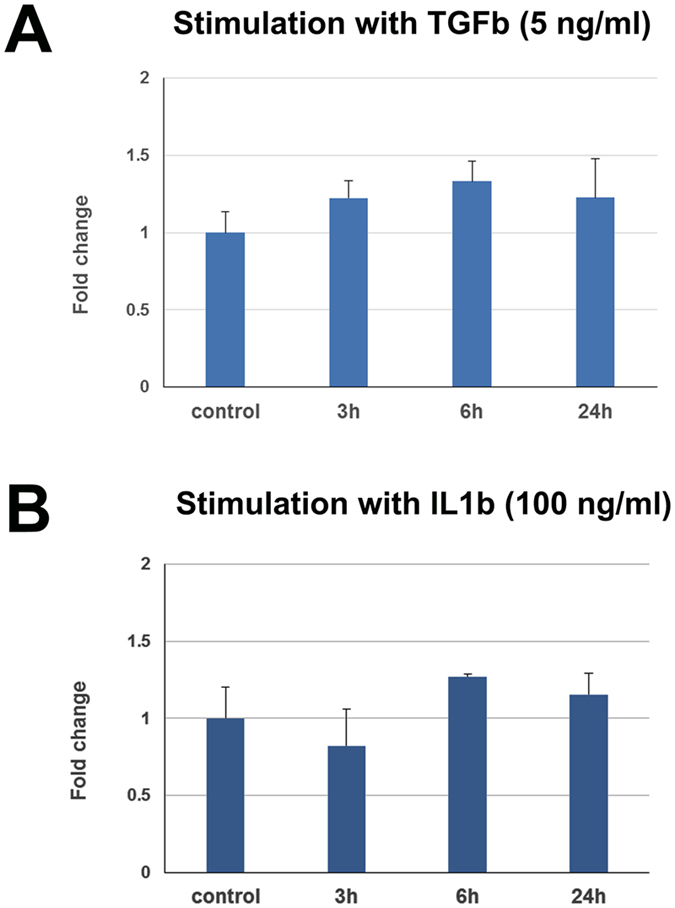
Expression profile of 3110045C21Rik after stimulation with TGFb and IL1b. (**A**) mRNA expression of 310045C21Rik after simulation with TGF-β1 (5 ng/ml) for 3, 6 and 24 hours. The mRNA levels were normalized to GAPDH and expressed as fold of induction/change to control experiment where no simulation took place. (**B**) mRNA expression of 310045C21Rik after simulation with IL1b (100 ng/ml) for 3, 6 and 24 hours. The mRNA levels were normalized to GAPDH and expressed as fold of induction/change to control experiment where no simulation took place.

Of special interest is the effect of *3110045C21Rik* overexpression on *Cdh1*, *Acta2* and *Tgfb1,* genes known to be involved in fibrosis. In most animal models (UUO, anti-GBM, ischemia-reperfusion) we recorded down-regulation of *3110045C21Rik* during the progress of fibrosis (Fig. 6B) while in the same conditions *Cdh1* is down-regulated and *Acta2* and *Tgfb1* are up-regulated. Interestingly when we overexpressed this lncRNA in cell lines, the expression levels of these genes were reversed. Taking into consideration that these changes are critical for the development of fibrosis it was interesting to check whether these can be induced by well-known factors of fibrosis. Therefore, we examined whether exogenous administration of TGFb and IL1b can induce these changes in the expression of *3110045C21Rik*. We provide evidence that both factors are not able to cause changes in the expression of this lncRNA (Fig. 8), suggesting that 3110045C21Rik may be critically involved in renal fibrosis, without being upstream regulated by TGFb of IL1b signaling.

## Discussion

Next generation sequencing techniques are useful means for detecting novel genes, transcriptional and epigenetic networks. These methodologies have recently been employed in common medical research; however, their use in nephrology is still very preliminary^32^,^33^ and they have not addressed so far the changes in the transcriptomic profile during the development of renal fibrosis.

To address this issue, we performed whole transcriptome analysis combined with bioinformatics analysis in the UUO mouse model of renal fibrosis. We present novel information regarding up-regulated and down-regulated genes, pathways and biological processes associated with the progression of fibrosis. Moreover, we provide lists of differentially expressed protein-coding genes, not suspected so far to be involved in the process, including many transcriptional regulators. We propose here that a subset of these factors may participate in gene regulatory networks involved in renal fibrosis. More specifically, we were able to show strong up-regulation of many transcription factors implicated in signaling and transcriptional cascades (Table 1, i.e. members of the AP-1 transcriptional cascade, Fosl1, Fosl2, Fos, Jun, JunD, Egr2 and Creb5). This pathway regulates the extracellular stimuli-mediated changes in gene expression program of many organs and diseases to mediate cellular changes related to inflammation, proliferation, adhesion, migration, apoptosis, wound healing, viral infections and neuronal plasticity^34^, raising the hypothesis that it may also be involved in transcriptional regulation during progression of renal fibrosis. Therefore, our data provide an initial map to facilitate the understanding of molecular changes in gene expression program that correlate with and mediate renal fibrosis. In a recent report, by Zhou *et al.*^35^ a transcriptomics analysis of Smad3 knock-out mice was performed in UUO injury. The comparison between their data and ours revealed similarities as well as interesting differences, probably due to different time points examined and analytical methodology used.

Thorough analysis of our data led to the association of a long list of non-coding RNA genes with UUO, not previously associated with renal pathology. Specifically, we showed that a large number of lncRNAs are expressed in renal tissue and exhibit altered expression patterns upon progression of renal fibrosis. The differential expression of these new molecular players in UUO model was firmly confirmed and established at the RNA and chromatin organization levels. Furthermore, we validated and documented the alterations in the expression profiles of selected lncRNAs in three other murine models of renal pathologies, and provided initial evidence for a putative functional role of this class of molecular regulators in renal cells. These observations add a new layer of molecular complexity in the understanding of renal fibrosis, not previously anticipated or considered.

In general, the new sequencing technologies and data from ENCODE and FANTOM have revolutionized our view of the organization, activity and regulation of the mammalian genome^36^,^37^,^38^, realising that the majority of the genome is transcribed producing vast numbers of formerly unknown regulatory lncRNA species^37^,^39^,^40^. LncRNAs are transcribed by RNApol II, can be post-transcriptional processed by 5′ capping, polyadenylation, splicing, RNA editing, and exhibit specific sub-cellular localization^41^. It was very recently shown that lncRNAs participate in the gene regulatory networks for the control of ESC pluripotency, proliferation and metastasis of cancer cells, as well as development and function of various tissues^42^,^43^,^44^. They have also been correlated with many human diseases and syndromes, either as biomarkers or causative factors^45^,^46^. In this respect, the list of novel lncRNAs uncovered in this study represents an important new source for potential regulators of renal pathologies. In the present report we focused on a limited number of lncRNAs that exhibited consistent transcriptional behaviour (up-regulation or down-regulation) in four different animal models of nephropathies, three of them of chronic nature, exhibiting fibrosis and one of acute nature, not associated with fibrosis. It is interesting that when tested on a tubular epithelial cell line, they all exerted different phenotypic alterations upon overexpression, suggesting that they can participate in a rather intricate pattern of regulatory networks during the development of nephropathies. However, it should be stressed that many other members of the identified lncRNAs might be even more interesting and possibly been involved in specific processes characteristic of special/exclusive aspects of each nephropathy.

In conclusion, our data from RNA-seq analysis of a murine model of renal fibrosis provide a list of novel and interesting sequences of coding and non-coding RNAs, which will allow us to better understand the complexity of the pathogenetic mechanisms encompassing but not limited to fibrosis and design more specific, targeted therapeutics in the near future.

## Methods

### Mouse model of UUO

Eight- to 12-week-old male C57BL/6 mice, were supplied from the colony of the Center of Experimental Surgery of our Institute. Mice underwent ligation of the right ureter and were divided into three groups during the surgery: sham operated, 2 days ligated and 8 days ligated. Before surgery the mice were anesthetized via face mask delivering sevoflurane^47^. The right ureter was exposed through a midline abdominal incision and was either completely obstructed 1 cm below the renal pelvis with 5.0 silk ligature (ligated animals) or manipulated similarly but not ligated (sham operated animals). Two or eight days after surgery the kidneys were collected, rinsed with isotonic saline, dissected and stored in liquid nitrogen for further analysis.

All aspects of animal experimentation were performed in adherence to the NIH and the European Union Guide for the Care and Use of Laboratory Animals and were approved by the Institutional Review Board and the Animal Experimental Committee of the Biomedical Research Foundation of the Academy of Athens.

### Other mouse models

The Renin overexpression model is a mouse model of hypertensive nephropathy, where *Ren-1* and *Ren-2* genes are inserted into a liver-specific locus. The resulting trans-gene expresses renin ectopically at a constantly high level and achieves elevated plasma levels of pro-renin, renin and angiotensin II throughout life. Sustained up-regulation of pro-inflammatory and pro-fibrotic macromolecules is observed, followed by high systemic blood pressure, leading to structural damage and functional decline of the kidney^48^,^49^,^50^.

The renal ischemia-reperfusion is a model of acute kidney injury, achieved by clamping the renal artery alone or the pedicle. The procedure usually involves ischemia induction from 15 to 60 min, followed by reperfusion; during the process, changes in coloration of renal parenchyma confirm the procedure’s effectiveness^51^.

The nephrotoxic serum-induced glomerulonephritis model is induced by injection of de-complemented heterologous serum containing immunoglobulins against glomerular antigens resulting in an acute inflammatory reaction characterized by immune cell infiltration. This model mimics the rapidly progressive glomerulonephritis in humans^52^,^53^.

### RNA isolation and cDNA synthesis

RNA was isolated from kidney tissues from all three animal groups using TRI reagent (Life Technologies, Carlsbad, CA). Sample integrity, quality and purity were determined accordingly. cDNA synthesis was performed from DNase1 treated RNA samples using ImProm-II Reverse Transcriptase (Promega Corp., Madison, WI) according to the manufacturer’s instructions.

### RNA-seq

Total RNA of all samples were sequenced by EMBL GeneCore (EMBL Genomics Core Facility, Heidelberg, Germany). The synthesis of polyadenylated transcriptome libraries for every sample was accompanied by deep sequencing in 3 lanes each generating >50 million reads pair end. The first two lanes comprised of 6 samples and the third of 4 samples.

### Real-Time PCR Analysis

Expression levels of protein coding genes known to be involved in renal fibrosis or cell cycle and selected lncRNAs were determined with RT-qPCR using SYBR Green and the Lightcycler 96 software (Roche). The reactions were performed using Platinum TaqDNA polymerase (Invitrogen), three-step standard cycling conditions, and sequence-specific primers designed (Supplemental Table 30).

Cycle conditions consisted of a pre-incubation step at 95 °C for 10 min, 45 cycles of 95 °C for 10 s, 60 °C for 15 s (plate reading), 72 °C for 15 sec. Melting curve analysis was included, with measurements taken every 1 °C from 65 °C to 95 °C. Experimental cycle thresholds (Cts) values were normalized to GAPDH Cts. Quantification of the levels of mRNA expression was done with the ΔΔCt method. The experiments were performed in triplicate and repeated at least three times. Data were expressed as the fold of induction in ligated animals relatively to sham operated animals ± SD and the analysis was performed using a t-test in Microsoft Excel (Redmond, WA).

### Bioinformatics analysis

#### Preprocessing

The FASTQ files containing pair-end sequence reads were mapped to the UCSC mm10 reference genome using tophat2^54^, with the standard parameters for reads obtained from mammalian sequencing with Illumina platforms, proposed by the authors. The–mate-inner-dist and–mate-std-dev tophat2 parameters, which are crucial for paired-end reads were estimated for each sample as follows: firstly, the FASTQ files were aligned to the mm10 transcriptome using bowtie2^55^. Then, sequence metrics were collected for the first 10 million properly mapped paired-end reads using Picard Tools^56^. Finally, from the resulting metrics that included average cDNA fragment size and its standard deviation, the aforementioned parameters were estimated by subtracting two times the short read length from the average fragment size, and using the standard deviation as-is respectively.

#### Functional and pathway analysis

Each list of differentially expressed genes derived from the different comparisons was subjected to functional and biochemical pathway analysis using the Gene Ontology^57^ and KEGG^58^ (Kyoto Encyclopedia of Genes and Genomes) and Panther^59^ biochemical pathway databases. The analysis was performed using the GeneCodis software^60^,^61^,^62^. Among the different GO structured networks we mainly focused on biological process and molecular function. Additionally, in order to define whether the differentially expressed genes were previously associated with renal fibrosis, extensive literature searches were performed using Pubmed and the Kidney and Urinary Pathway Knowledge Base (kupkb database)^12^.

#### Differentially expressed genes

Differential gene expression analysis was performed with the Bioconductor package metaseqR^63^. Briefly, sequence reads were counted over mm 10 exons and after filtering, the final read counts for each gene were calculated as the sums of their exon reads, creating a gene counts table where each row corresponded to an Ensembl gene model and each column corresponded to an RNA-seq sample. The gene counts table was normalized using DESeq^64^ after removing genes that had zero counts over all the RNA-seq samples. Prior to the statistical testing procedure, the gene read counts were filtered for possible artifacts that could affect the subsequent statistical testing procedures. Genes presenting any of the following were excluded from further analysis: i) genes with length less than 500, ii) genes whose average reads per 100 bp was less than the 25^th^ quantile of the total normalized distribution of average reads per 100 bp, iii) genes with read counts below the median read counts of the total normalized count distribution. The resulting gene counts table was subjected to differential expression analysis for the contrasts SO versus 2D, SO versus 8D and 2D versus 8D, using DESeq.

The data discussed in this manuscript have been deposited in NCBI’s Gene Expression Omnibus^10^,^11^ and are accessible through GEO Series accession number GSE79443. (https://www.ncbi.nlm.nih.gov/geo/query/acc.cgi?acc=GSE79443).

#### **Chromatin immunoprecipitation (ChIP)**

ChIP was performed as previously described^65^,^66^. Immunoprecipitation was performed with antibodies against RNA polymerase II (Millipore, Billerica, MA) and Histone 3 (tri-methylated K4) (ab8580, Abcam, Cambridge, England) and a normal rabbit IgG (sc- 2027, Santa Cruz Biotechnology, Inc, Germany) was used as a control. Precipitated DNAs were detected by PCR using primers for the promoter regions of lncRNAs (Supplemental Table 31).

All experiments were performed in triplicate and repeated at least three times. Statistical analysis was performed by the paired two-sample Student’s t test.

#### **Cell culture**

Mouse kidney epithelial cells (M-1 cell line) were purchased from the American Type Culture Collection (ATCC, CRL-2038). The cell line was established from normal renal tissue taken from a mouse transgenic for the SV40 early region (tg(SV40E)Bri7). The cells retain many characteristics of cortical collecting duct (CCD) cells including morphology and antigens.

Cells were cultured at 37 °C in a humidified atmosphere of 95% air/ 5% CO_2_, and maintained in a 1:1 mixture of Dulbecco’s modified Eagle’s medium and Ham’s F12 medium with 2.5 mM L-glutamine (1:1 DMEM/F12) adjusted to contain 15 mM HEPES, 0.5 mM sodium pyruvate and 1.2 g/L sodium bicarbonate supplemented with 100 U/ml penicillin, 100 μg/ml streptomycin and 5% fetal bovine serum. Cells were subcultured using trypsin–EDTA at a ratio of 1:3 to 1:4 as recommended by the manufacturer.

#### **Preparation of plasmid constructs for lncRNAs**

Plasmid constructs of three selected lncRNAs (RP23-45G16.5, 3110045C21Rik, AI662270) were manufactured by GenScript (Piscataway, USA). The sequence of the lncRNAs was provided by the authors and the plasmids were constructed using vector pcDNA3.1(+) as backbone and KpnI-NotI as cloning site in all three cases.

#### **Transient transfection and stimulation of M-1 cell line**

For the purpose of transfection and stimulation, cells were plated at 6-well plates. During transfection, plated cells were transfected with plasmid constructs of the 3 selected lncRNAs or empty vector, (the backbone used to synthesize the lncRNAs plasmids constructs) using the lipofectamine 2000 reagent (Invitrogen Corp., Carlsbad, CA). The evaluation of the lncRNAs overexpression was performed by real-time RT-qPCR. The stimulation experiments were performed following 24-hour serum deprivation. During stimulation, plated cells were stimulated with TGF-β1 (5 ng/ml), IL1b (100 ng/ml) or vehicle for 3, 6 and 24 h in serum-deprived conditions. Each experiment and/or time point was followed by total RNA extraction and cDNA synthesis performed as previously described. All experiments were performed in triplicate at least three times and statistical analysis was performed by the paired two-sample Student’s t test.

#### **Proliferation assay**

Cell proliferation was studied using a BrdU (bromo-deoxy-uridine)-incorporation immunolabeling assay as previously described^67^ after co-transfection of the cells with a GFP construct and lncRNAs constructs at a ratio 1:3.

An anti-BrdU primary antibody (ab6326; Abcam) was used. To detect the incorporated BrdU, a fluorescent secondary antibody (anti-rat Alexa Fluor; Invitrogen) was used. Cells were counterstained with the fluorescent dye DAPI for nuclei visualization and mounted on slides using a special fixative agent (DAKO).

A fluorescence microscope (DMRA2; Leica Microsystems GmbH, Wetzlar, Germany) was used to examine cells and random non-overlapping images were taken at 200X magnification. Results were expressed as percentage of the ratio of BrdU measurements (proliferating cells) to GFP measurements (efficiently transfected cells). Each experiment was performed in triplicate and was repeated at least three times.

## Additional Information

**How to cite this article**: Arvaniti, E. *et al.* Whole-transcriptome analysis of UUO mouse model of renal fibrosis reveals new molecular players in kidney diseases. *Sci. Rep.* **6**, 26235; doi: 10.1038/srep26235 (2016).

## Supplementary Material

Supplementary Dataset

Supplementary Information

## Figures and Tables

**Table 1:** Transcription factors or regulators not previously assosiated with renal fibrosis.

Gene	Fold Change SO vs 2D	Log2 Fold Change SO vs 2D	p-value	Fold Change SO vs 8D	Log2 Fold Change SO vs 8D	p-value
Sox9	55.131	5.785	6.49E-35	50.395	5.655	8.55E-34
Creb5	13.935	3.801	4.41E–05	8.946	3.161	0.000499581
Pou1f1	13.010	3.702	0.003193411	14.020	3.809	0.002526991
Uhrf1	11.428	3.514	6.08E–16	11.529	3.527	4.94E–16
Mis18bp1	9.116	3.188	5.73E–07	4.713	2.237	0.000308533
Sox11	8.000	3.000	0.000273125	25.446	4.669	1.09E–07
E2f7	7.649	2.935	0.000566974	6.717	2.748	0.001167639
Lhx2	7.459	2.899	0.047260521	9.369	3.228	0.029423642
Foxj1	7.267	2.861	2.71E–10	35.513	5.150	2.12E–25
Relb	6.574	2.717	1.50E–11	12.314	3.622	2.78E–18
Bcl3	6.302	2.656	3.38E–11	5.658	2.500	3.64E–10
Arid5a	6.044	2.596	4.64E–09	8.662	3.115	4.59E–12
Hells	5.929	2.568	8.51E–07	4.554	2.187	2.28E–05
Brca1	5.916	2.565	2.20E–05	3.772	1.915	0.001278627
Hesx1	5.857	2.550	0.0001854	Not statistically significant in this comparison		
Fosl1	5.727	2.518	9.26E–05	6.357	2.668	3.71E–05
Foxm1	5.645	2.497	9.10E–08	8.384	3.068	1.16E–12
Maff	5.530	2.467	5.69E–09	18.200	4.186	1.06E–18
Runx1	5.072	2.343	1.42E–07	7.784	2.961	9.10E–07
Cdca7	5.071	2.342	7.62E–05	3.619	1.856	0.002473687
Asf1b	4.608	2.204	0.000360743	14.394	3.847	2.99E–06
Batf	4.413	2.142	0.005937201	21.757	4.443	9.04E–17
Egr2	4.089	2.032	3.81E–05	3.130	1.646	1.30E–05
Atf5	3.976	1.991	1.61E–07	4.047	2.017	9.87E–08
Sbno2	3.914	1.969	1.92E–07	3.544	1.825	0.001291195
Etv4	3.444	1.784	0.001644233	3.173	1.666	0.000280941
Arntl2	3.220	1.687	0.004706731	5.134	2.360	9.80E–05
Fosl2	3.200	1.678	1.04E–05	5.219	2.384	7.08E–10
Ciita	2.933	1.552	0.000500544	6.066	2.601	1.23E–08
Zfp182	2.735	1.452	0.001031867	Not statistically significant in this comparison		
Elf3	2.734	1.451	0.000111241	4.097	2.035	8.19E–08
Sox4	2.553	1.352	0.000870541	5.374	2.426	5.02E–09
Ezh2	2.330	1.220	0.006650586	2.018	1.013	0.023999984
Ifi205	2.313	1.210	0.003867694	12.666	3.663	2.19E–16
Bex1	2.303	1.203	0.019004634	2.199	1.137	0.026564847
Ikzf4	2.285	1.192	0.018368417	Not statistically significant in this comparison		
E2f1	2.280	1.189	0.004909231	1.891	0.919	0.029286962
Chaf1a	2.210	1.144	0.021923246	Not statistically significant in this comparison		
Rbpms	2.105	1.074	0.003833858	2.414	1.272	0.000631062
Hmgb2	2.063	1.045	0.005585089	2.762	1.466	0.000108467
Tgif1	2.038	1.027	0.006779508	3.099	1.632	1.95E–05
Zfp57	1.895	0.922	0.016739514	2.943	1.557	0.002821029
