# Supplementary material for: Whole-transcriptome analysis of UUO mouse model of renal fibrosis reveals new molecular players in kidney diseases
Source: Sci Rep. 2016 May 18;6:26235. doi: 10.1038/srep26235 (PMC4870569; doi:10.1038/srep26235)
Supplement: Supplementary Information [file srep26235-s2.pdf]

Whole-transcriptome analysis of UUO mouse model of renal fibrosis reveals new molecular players in kidney diseases

Eleni Arvaniti[1], Panagiotis Moulos[2], Athina Vakrakou[1], Christos Chatziantoniou[3], Christos Chadjichristos[3], Panagiotis Kavvadas[3], Aristidis Charonis[1*] and Panagiotis K Politis[4*]

[1]*Center for Clinical, Experimental Surgery and Translational Research, Biomedical Research Foundation of the Academy of Athens, Athens, Greece;*

[2]*HybridStat Predictive Analytics, Athens, Greece;*

[3]*INSERM UMR-S1155, Tenon Hospital, Paris, France;*

[4]*Center for Basic Research, Biomedical Research Foundation of the Academy of Athens, Athens, Greece.*

* these authors should be considered as last co-authors

Corresponding author:

Aristidis S. Charonis M.D. Ph.D.

Center for Clinical, Experimental Surgery and Translational Research,

Biomedical Research Foundation of the Academy of Athens,

Soranou Efessiou 4, Athens 115 27, GREECE

tel. +30.210.6597205

fax. +30.210.6597545

e-mail: acharonis@bioacademy.gr

SUPPLEMENTAL FIGURE LEGENDS

Supplemental Figure 1

Immunofluorescence of the renal parenchyma with Mac-2 staining, **A.** 2 days and **B.** 8 days after ligation. An increase in staining is observed with the progression of the fibrosis.

Supplemental Figure 2

ChIP analysis for the binding of RNA polymerase II (RNA pol) and H3K4m3 in the promoter region of selected lncRNAs. For these experiments chromatin samples were prepared from kidneys of sham operated (SO), 2 days ligated (2D) and 8 days ligated (8D) mice.

Supplemental figure 3

Evaluation of the overexpression of lncRNAs in transfection experiments, performed by RT-q-PCR. A. RP24-45G16.5 RNA, B. 3110045C21Rik RNA and C. AI662270 RNA. The RNA levels of each lncRNA were normalized to GAPDH and expressed as fold of induction/change compared to control cells transfected with empty vector.

Immunofluorescence with anti-Mac2 antibody

A

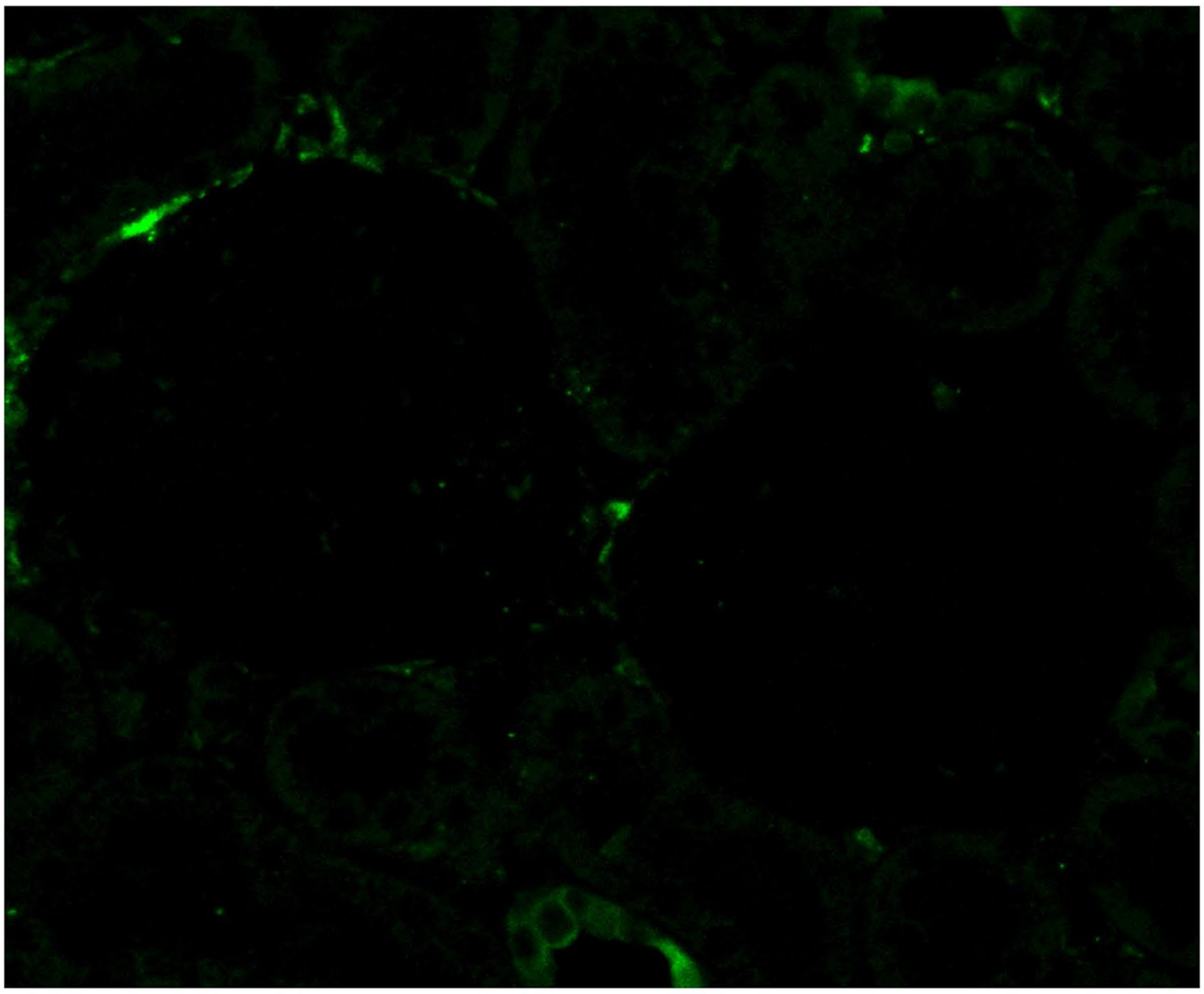

2D

B

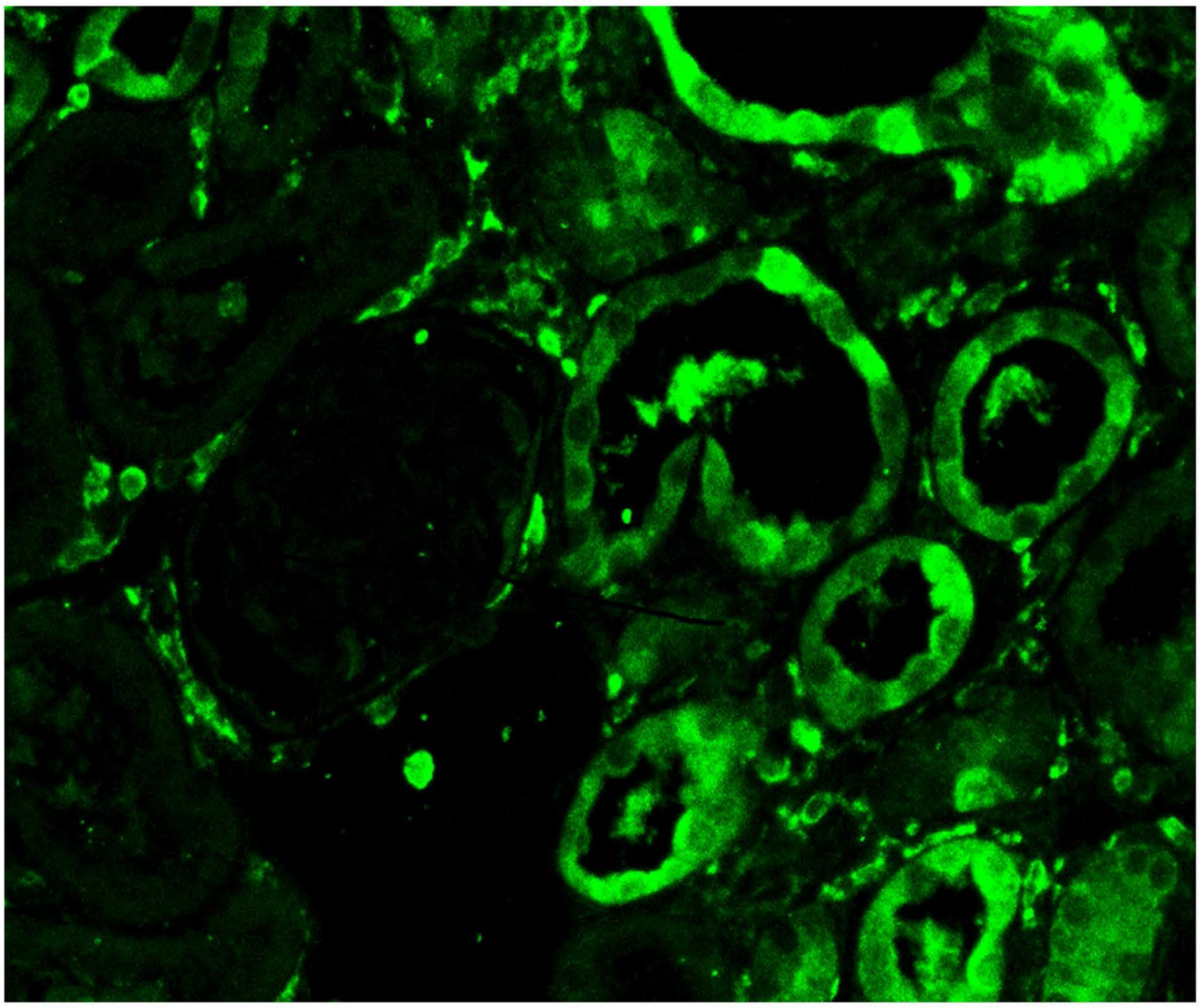

8D

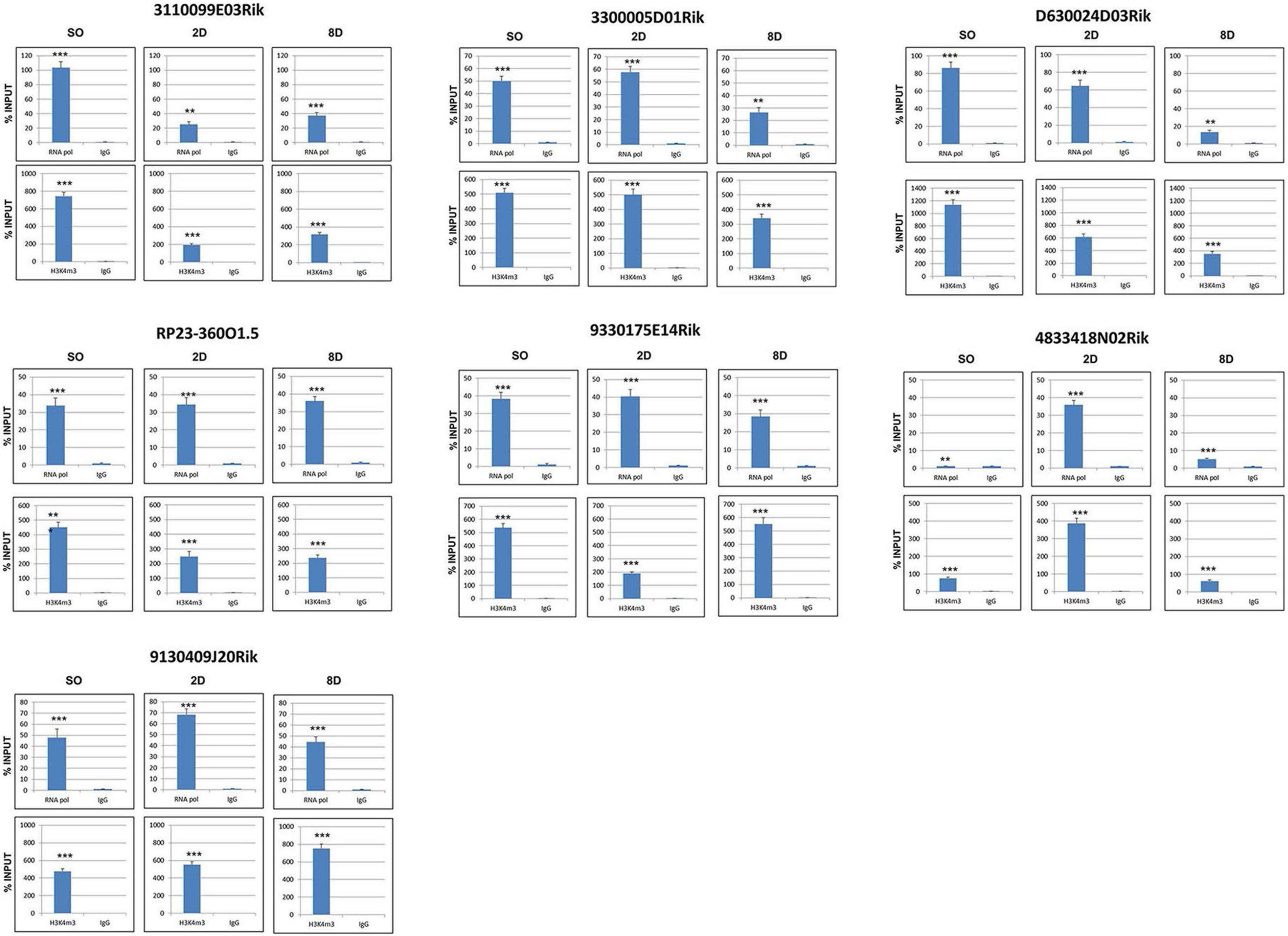
3110099E03Rik
3300005D01Rik
D630024D03Rik
RP23-360O1.5
9330175E14Rik
4833418N02Rik
9130409J20Rik
SO
2D
8D
% INPUT
RNA pol
IgG
H3K4m3

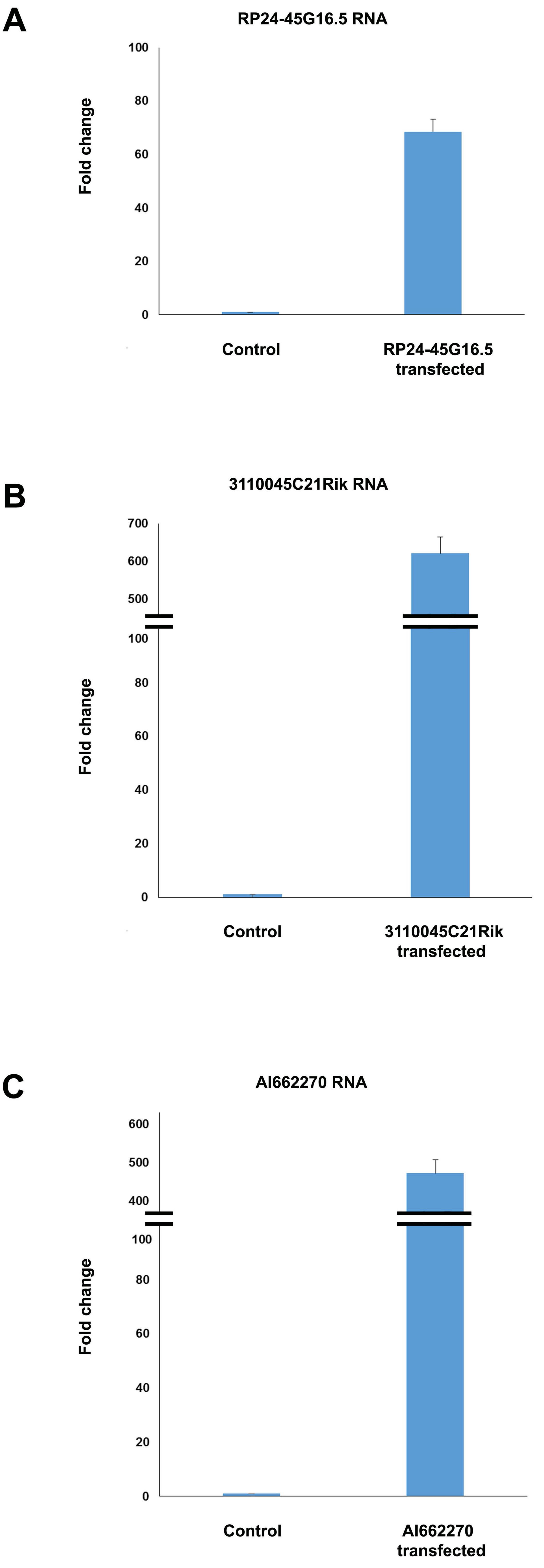
A
RP24-45G16.5 RNA
Fold change
100
80
60
40
20
0
Control
RP24-45G16.5
transfected
B
3110045C21Rik RNA
Fold change
700
600
500
100
80
60
40
20
0
Control
3110045C21Rik
transfected
C
AI662270 RNA
Fold change
600
500
400
100
80
60
40
20
0
Control
AI662270
transfected
